# Radiation and Glands

**Published:** 1905-12-02

**Authors:** 


### RADIATION AND GLANDS.

(*Continued from page* 135.)

There is evidence to show that this depressing influence of the *x*-rays upon glands has had an untoward result upon the sexual organs of persons of both sexes who have been exposed for considerable periods. Tilden Brown [9] and Osgood have discovered that the examination of the seminal fluid in 16 persons, makers of tubes or men who have been exposed during their treatment of patients, reveals the fact that either the spermatozoa are absent, or there is a condition of oligo-necrospermia. As they remark, it will take a long time before the actual cause of this can be ascertained, whether it be a particular kind or quality of the rays, the intensity of the dose, or the length of, or repetition of, the exposures. It seems that repeated prolonged exposures may do so, but there is as yet no evidence to show whether the condition is a permanent one or not. Margaret Cleaves,[10] in commenting upon this report, quotes a case where a patient had been treated for pruritus ani with the *x*-rays, with a result that active spermatozoa, which were known to have been present before treatment, disappeared for some months afterwards; after some three months there was a gradual return to the normal, and active spermatozoa could again be discovered. Frieben found that in animals the epithelium of the seminal tubules disappeared after exposure. Halberstaedter[11] has found that profound macroscopic and microscopic changes occurred in the ovaries of rabbits under the influence of *x*-rays, the most evident histological change being the complete disappearance of the Graafian follicles. These experiments were controlled by preliminary laparotomies for the inspection of the ovaries, and the subsequent protection of the ovary on one side. Only the organ on the unprotected side presented the changes. It seemed that the ovaries were more sensible to the rays than the skin of the abdomen. Less time and fewer exposures were necessary to produce corresponding changes in the organs of male rabbits. Cleaves proceeds to discuss the observation that fibroids of the uterus have diminished in size under the rays, questioning whether this may not be secondary to changes in the ovaries. While deducing from these reports the urgent need for more efficient protection of those working with rays, and of patients who are exposed, she raises the question of the use of the *x*-ray as a conservative agent in preference to the surgeon's knife.

With regard to the action of the rays in retarding development, some interesting experiments are recorded by Bordier and Galimard.[12] Gilman and Baetjer have studied the development of hens' eggs under the rays. Using 12 eggs, they set aside six as controls and incubated them. Six more were exposed to a known quantity of the rays (15 H) before incubation, and in all 14 exposures similar to the first were given. After 20 days' incubation a control was opened, and showed a chick at term: in one that had been rayed the white and yellow came out separately. Two days later all were opened, and it was found that not one of those rayed showed the least sign of commencement of embryonic formation. Eggs incubated at first, and later exposed to the rays, showed development up to the point at which radiation was begun. One egg obtained one dose of 15H, and this showed arrested development from that time onwards. The albumen of the rayed eggs showed changes, in that it was much less viscid and coagulated with difficulty. When coagulated it showed delay in digestibility in artificial gastric juice. There was no sign of decomposition.

[9] Archiv. of Rönt. Ray. March 1905. [10] Med. Elect. and Radio., March, 1905. [11] Berliner Klin. Woch., January 16, 1905 (quoted by Cleaves). [12] Med. Electro. and Radio., July, 1905.

